# New strategy for COVID-19 vaccination: targeting the receptor-binding domain of the SARS-CoV-2 spike protein

**DOI:** 10.1038/s41423-020-00584-6

**Published:** 2020-11-26

**Authors:** Junzhi Wang

**Affiliations:** Key Laboratory of the Ministry of Health for Research on Quality and Standardization of Biotech Products, National Institutes for Food and Drug Control, Beijing, China

**Keywords:** Vaccines, Infectious diseases

**Fig. 1:**
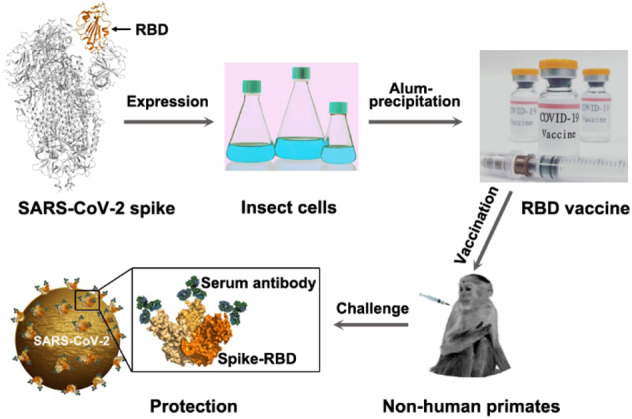
A recombinant vaccine targeting the receptor-binding domain (RBD) of the SARS-CoV-2 spike can induce a protective immune response. The recombinant RBD protein was expressed in insect cells using the Bac-to-Bac system and Alum-precipitated for vaccine formulation. After the vaccination of the nonhuman primate monkeys with the RBD vaccine, serum antibodies that could bind the RBD and neutralize viral infection were effectively induced, providing the rationale for the development of a protective vaccine through the induction of antibodies against the RBD

The unexpected outbreak of SARS-CoV-2 infection, with over 42.5 million confirmed cases and more than 1.1 million deaths globally,^1^ has posed a great threat to public health. Despite global efforts to control this viral infection, the pandemic is still surging. While facing such a serious, unprecedented situation, vaccines are likely the most promising method to control this newly identified coronavirus. A recent study from Yang et al.^2^ showed that a recombinant spike receptor-binding domain (RBD) protein of SARS-CoV-2 prepared from insect cells induced protective immunity in nonhuman primates, providing a feasible vaccine candidate for COVID-19 control (Fig. 1).

To establish an infection, SARS-CoV-2 needs to enter host cells by first engaging the cellular receptor of angiotensin-converting enzyme II (ACE2).^3^ This receptor recognition process is mediated by viral envelope-embedded spike trimers. Both structural and functional data demonstrate that the RBD region of the spike specifically interacts with ACE2.^4^ Antibodies that target the RBD and interfere with spike binding to ACE2 would block viral entry and thereby neutralize viral infection. Thus, the spike RBD is a good immunogen for vaccine development. In recognition of the indispensable role of the spike RBD in SARS-CoV-2 infection, Yang et al. selected the spike region spanning residues 319-545 as a candidate immunogen. This spike region, according to the solved, complex structure of the SARS-CoV-2 RBD bound to ACE2,^4^ represents an integral structural entity responsible for receptor engagement.

To ensure that the spike RBD protein could be effectively expressed and that the expressed protein maintained its native conformation, Yang et al. selected the Bac-to-Bac Baculovirus Expression system for immunogen preparation. As one of the most powerful and versatile expression systems available, the Baculovirus Expression Vector System (BEVS) is a helper-independent viral system that as developed to express heterologous genes in insect cells. The Bac-to-Bac Baculovirus Expression System is a modified BEVS in which a recombinant Baculovirus containing the foreign gene is first generated and then used to infect insect cells for high-level expression of genes from many different sources. In comparison to other heterologous expression systems, Bac-to-Bac has several advantages. It has a large cloning capacity for foreign genes, is easily screened for recombinant Baculoviruses, is highly secure, possesses a complete posttranslational processing and modification system, and in most cases, can guarantee the effective expression of the gene of interest. Since its discovery in the 1980s,^5^ BEVS has been widely used in vaccine development, gene therapy and other fields. As expected, the SARS-CoV-2 spike RBD was properly expressed in insect cells using the Bac-to-Bac system. The subsequently prepared RBD protein was both N- and O-glycosylated and fully functional, interacting with ACE2 with a nanomolar binding affinity. In addition, the recombinant protein specifically reacted with the convalescent sera of SARS-CoV-2-infected patients but not with the sera of healthy individuals.

Can the RBD protein induce an effective immune response? To settle this issue, Yang et al. immunized both mice and rabbits with the recombinant RBD protein using aluminum hydroxide as an adjuvant. The Alum-precipitated protein was shown to induce a robust humoral immune response. RBD-specific antibodies were generated in both animals. Furthermore, the sera from those immunized animals were observed to block RBD binding to ACE2 expressed on the cell surface and to neutralize the viral infection of both the pseudotyped and live SARS-CoV-2 in vitro. More importantly, a functional antibody response was observed as early as 7 days after the injection of a single dose, suggesting that the recombinant RBD protein is highly antigenic.

As a key step towards vaccine development, Yang et al. further tested the immunogenic effect of the RBD vaccine in nonhuman primates (*Macaca mulatta*). The immune sera collected 7 days after the first vaccination effectively blocked infection by SARS-CoV-2 pseudovirus. Neutralizing antibodies against live virus were detected in all vaccinated monkeys after two intramuscular injections of the RBD vaccine. Following a challenge with live virus, no detectable viral genomic RNA (gRNA) or subgenomic RNA (sgRNA, indicative of viral replication) were observed in lung tissues in the vaccinated groups. In addition, significant histopathological differences in the lung tissue were observed between control and vaccinated nonhuman primates. While the unvaccinated animals all showed typical histopathological changes of viral interstitial pneumonia (as exemplified by thickened alveolar walls, large amounts of interstitial mononuclear inflammatory cell infiltrates, and the disappearance of recognizable architecture), monkeys that received the RBD vaccine exhibited no significant histopathological changes, largely featuring the appearance of normal lung tissues.

Taken together, the data reported by Yang et al. clearly demonstrate that the recombinant spike RBD protein of SARS-CoV-2 can potently induce a protective immune response in mice, rabbits, and nonhuman primates and thus warrants further investigation for clinical trials in humans. Yang’s results also highlight the importance of the spike RBD in SARS-CoV-2 vaccine design and provide a vaccine candidate to counteract COVID-19.
